# Customer Abuse and Aggression as Labour Control Among LGBT Workers in Low-Wage Services

**DOI:** 10.1177/09500170211045843

**Published:** 2021-11-01

**Authors:** Suzanne Mills, Benjamin Owens

**Affiliations:** McMaster University, Canada; McMaster University, Canada

**Keywords:** customer abuse, discrimination, gender, heteronormativity, labour control, labour process theory, LGBT, service sector, transgender

## Abstract

This study examines the relation between customer abuse and aggression, the gender and sexual expression of workers, and labour control in low-wage services. In-depth interviews with 30 lesbian, gay, bisexual, and transgender (LGBT)^1^ low-wage service sector workers reveal how customer abuse and aggression works in consort with management strategies to reproduce cis- and heteronormativity. Customer abuse and aggression disciplined worker expressions of non-normative gender and sexual identities, leading to concealment and self-policing. Management was complicit in this dynamic, placing profitability and customer satisfaction over the safety of LGBT workers, only intervening in instances of customer abuse and aggression when it had a limited economic impact. It is posited that customer abuse and aggression is not only a response to unmet expectations emanating from the labour process but is also a mechanism of labour control that disciplines worker behaviour and aesthetics, directly and indirectly, by influencing management prerogatives.

## Introduction

Low-wage service work has expanded over the past 50 years, now often rivalling employment in goods producing sectors in the labour markets of industrialized countries.^2^ A sizable body of research has described the distinct attributes of this work, examining how customer interaction affects the labour process and the wellbeing of workers (Lopez, 2010). In particular, the type of labour required and the mechanisms used to control it are shaped by the centrality of customer satisfaction to profitability. Workers in low-wage services are often expected to engage in emotional and aesthetic labour which is gendered and sexualized (Hochschild, 1983; Warhurst and Nickson, 2009). Moreover, customers play a contingent role in labour control, at times reinforcing or mystifying management efforts, or exerting more direct control in others (Gamble, 2009). The central role of customers in the labour process and the gendered nature of service work often coalesce to render low-wage service workers vulnerable to customer abuse and aggression with negative consequences for their wellbeing (Korczynski and Ott, 2004).

Previous scholarship has theorized customer abuse and aggression as an outcome of the labour process and worker identity. Customer abuse and aggression is understood to result from the power imbalance between customers and workers and the contradiction between rationalization and customer sovereignty (Boyd, 2002; Korczynski and Ott, 2004). This power imbalance is exacerbated if workers are from identity groups of weakened social status vis-a-vis customers, increasing the prevalence of abuse (Korczynski and Evans, 2013). Several scholars have also drawn attention to how the gendered labour process in low-wage services contributes to customer abuse and aggression. Both the emotional labour required and the gendered commodification of workers’ bodies and identities as part of the service experience—particularly in workplaces where sexualized performances of female heterosexuality are expected—renders women workers particularly vulnerable to verbal, physical and sexual harassment (Filby, 1992; Forseth, 2005; Guerrier and Adib, 2000; Warhurst and Nickson, 2007; Waring, 2011).

In these conceptualizations, customer abuse and aggression in low-wage services is the result of a labour process that creates an illusion of customer sovereignty, and/or fosters gendered customer expectations. Customers are therefore reacting to, rather than acting on, the labour process when they engage in abuse. Locating the genesis of customer abuse in the labour process helps to explain the customer abuse faced by women and low-wage service workers as a whole. It does not, however, adequately account for the ways that customer actions might, in both intent and effect, influence worker behaviour and labour, particularly it’s gendered aesthetic and emotional dimensions.

Research about LGBT workers has found that this group often faces heightened harassment and discrimination from co-workers, employers and customers, and that their experiences can expose taken-for-granted gender norms embedded in the labour process (Denissen and Saguy; Willis, 2009). This is particularly apt in low-wage services where LGBT workers are overrepresented (Waite et al., 2020; Whittington et al., 2020). Unpacking LGBT workers’ experiences of customer abuse can therefore usefully advance understandings of the complex ways that gender and sexual norms are reproduced through customer–management–worker interactions in low-wage services. Non-heterosexual sexual orientations and transgender identities challenge traditional binary understandings of gender since the performance of heterosexuality and cisnormativity are socially understood to be part of what makes someone feminine or masculine; in the realm of discrimination then, sexual orientation therefore cannot be fully disentangled from gender (Valdes, 1996).

This research draws on the experiences of LGBT workers to better understand the relationship between customer violence, gender and sexual expression, and labour control in the low-wage service sector. The first section brings scholarship about customer abuse into conversation with scholarship about the role of customers in labour control to argue that customer abuse can be an influential form of labour control, particularly in the realm of gender expression. This is followed by a brief discussion about how LGBT worker experiences can inform understandings of gender in service sector workplaces. The article then draws on in-depth interviews with LGBT workers in two small cities to show how customer abuse and aggression worked both directly and indirectly with management to control the gender and sexual expression of workers. This research fills two lacunae in current scholarship: first, much of the research in interactive services examines binary conceptions of gender and heterosexual forms of sexuality and, as a result, has not fully explored how the complexity of gender and sexuality factors into the service interaction; second, scholarship has scarcely explored how customer abuse and aggression is not only a response to the labour process, but also a form of labour control that plays a role in reproducing and defining gendered hierarchies and norms.

### Customer abuse and labour control in the interactive services

The role customers play in the labour process of customer-orientated services has attracted a significant amount of scholarly attention over the past three decades. Central to this scholarship is Leidner’s service triangle (1993, 1999), which theorizes the labour process in interactive services as a triadic set of relationships, where workers, managers and customers all vie for power and control. For Leidner, control over the labour process is contingent, arising from how this constellation of relationships is realized in specific workplace and situational contexts. Relatedly, Korczynski’s (2003) notion of the ‘customer-oriented bureaucracy’—a term used to describe the organization of service work to simultaneously focus on customer service and a rationalized labour process—posits that workers are controlled by ‘customer-related norms and bureaucratic measurement’, creating a fragile situation ‘such that the irate, abusive customer becomes an important social actor’ (Korczynski, 2007: 578). Notwithstanding these approaches, scholars exploring customer abuse have tended to position management, and not customers, as setting the stage for workers’ experiences of abuse.

Korczynski’s (2002) influential theorization of customer abuse hinges on how the service triangle renders workers vulnerable to abuse and aggression by creating a dual logic where workers need to be efficient (to ensure production) and provide attentive customer service. In pursuit of profit through customer satisfaction and rationalization, management creates an illusion of ‘customer sovereignty’ where ‘it appears to the customer that he/she is sovereign, while at the same time creating space for the frontline worker to guide the customer through the constraints of production’ (Korczynski and Ott, 2004: 581). To uphold this illusion, a power imbalance is embedded into the labour process that renders workers subservient to customers yet powerless to defend themselves (Bishop and Hoel, 2008; Hughes and Tadic, 1998). Customer abuse and aggression is posited as a consequence of this contradiction between rationalization and a service environment in which ‘the customer is always right’: when the illusion of sovereignty is dispelled and customers’ expectations of service quality are not met, customers can become frustrated and aggressive (Bishop and Hoel, 2008; Boyd, 2002; Korczynski and Ott, 2004; Yagil, 2008). Building on this work, Korczynski and Evans (2013) theorized three additional factors that influence workers’ vulnerability to customer abuse and aggression: the degree of power a worker has (either through skill or collective representation), whether the customer interaction is repeated over time and whether workers have higher or lower social status than the customer.

Similarly, research exploring the gendered, racialized and sexualized nature of many service workplaces has emphasized how the infusion of gendered and sexualized aesthetics in the workplace by management can instigate customer harassment of women workers (Guerrier and Adib, 2000; Hughes and Tadic, 1998; Kern and Grandey, 2009). The gendered aesthetic requirements of different workplaces often require distinct gendered performances of femininity or masculinity, as well as heterosexuality (Warhurst and Nickson, 2009). When value is placed on the aesthetic dimension of the service interaction and workers’ bodies and identities are commodified as part of this aesthetic, customers may feel that they are getting a lower value service if workers do not meet their expectations (Warhurst and Nickson, 2007; Waring, 2011). Just as a failure to meet expectations of service quality can lead to customer abuse and aggression, so too can a failure to meet aesthetic and emotional expectations; as Yagil (2008: 144) explains in a review on the antecedents of customer abuse, ‘service organizations promote exaggerated images of the service, resulting in inevitable disappointment’ (Yagil, 2008). High levels of sexual harassment in the sector are therefore the result of the ways that the labour process commodifies workers’ bodies. To the extent that ‘the irate and abusive customer should be seen as a systemic part of the social relations of service work’ (Korczynski, 2003: 57), however, there has been less attention on how this abuse and aggression shapes the labour process, save for Korczynski’s (2003) suggestion that it leads to communities of coping as a form of worker resistance. What is more, few if any studies have linked customer abuse and aggression to customers’ attempts to exert control over the labour process.

A separate but overlapping body of scholarship has sought to account for the multiple ways that customers participate in labour control and resistance (Bélanger and Edwards, 2013; Korczynski, 2007; Lopez, 2010). Though employers might oppose customer control of workers in some instances because it conflicts with managerial desires for rationalization, perhaps more frequently employers seek to enlist customers in their labour control efforts (Gamble, 2009).

Using customers to bolster labour control is often desirable because of the centrality of customer satisfaction to profitability and the difficulty of monitoring the emotional and aesthetic aspects of work (Du Gay and Salaman, 1992; Fuller and Smith, 1991; Gamble, 2007). Customer control is particularly effective as a form of normative control since a fear of conflict with customers and the desire to maintain peace can create an internalized responsibility for service quality, influencing how workers comport themselves and leading them to self-police (Carls, 2009; Fuller and Smith, 1991; Gamble, 2007; Lloyd, 2017). Customer feedback mechanisms—either solicited by management or initiated by customers themselves—is used to monitor and discipline workers for their ‘attitudes and overall presentation of self’ or to identify exemplary workers for rewards (Fuller and Smith, 1991; Gamble, 2007). In these accounts, customer control is not powerful in isolation, but is rather a tool used to bolster management control (Fuller and Smith, 1991). Johnston and Sandberg (2008: 412) provide a slightly different account, demonstrating how customers vie for control over the labour process more directly alongside management and workers, which is then accommodated by management—for instance, when management defers to customers’ demands over the established rules of the workplace—in order to ‘sustain the service encounter’.

This article understands customer abuse as not only arising from the service labour process, but also as helping to shape it. As will be developed below, through acts of abuse and aggression, customers influence worker behaviour whether the consequences of these attempts align with those of management and augment the profitability of the firm or not (Du Gay and Salaman, 1992; Gamble, 2007). This understanding of customer abuse and aggression as playing a role in labour control has not yet figured into the literature on customer abuse and aggression, nor have the experiences of LGBT service workers.

### LGBT workers in interactive services

LGBT workers’ experiences of interactive service work are near-absent from the literature, yet they have the potential to provide unique insights into the service labour process. Paradoxically, cross-sectoral studies have often depicted low-wage services as both a refuge for LGBT workers and as increasing their vulnerability to abuse (David, 2015; Ozturk, 2011). In Turkey, Ozturk (2011) found that jobs in ‘retail, entertainment and tourism’ were the only sectors where LGB interviewees stated that they would not be fired. The clustering of gender and sexual minorities into some service sector jobs also led David (2015) to coin the term ‘purple collar’ to describe the overrepresentation of transgender women in call centres in the Philippines—workplaces that allowed transgender women to be ‘out’ yet also confined their gender performance to that desired by management. While not examining customer abuse and aggression explicitly, these studies connect these overt and subvert requirements to conform to gender norms to abuse from co-workers, managers and customers.

Research about LGBT workers in other sectors highlights how heteronormativity and cisnormativity operate in the workplace. Abuse and aggression—ranging from microaggressions, to misgendering and impeded bathroom access, to physical assault from co-workers and managers—often arises when workers challenge normative gender and sexual norms (Collinson and Collinson, 1989; Griffin, 2008; Schilt and Connell, 2007). A central contribution of this work is that workplaces are therefore not asexual places of production, but rather are imbued with heteronormativity and cisnormative gender hierarchies. When workers fail to conform to gender and sexual expectations, they often experience hostility from others who view non-conformity as a challenge not only to heteronormativity, but to the hierarchy it upholds (Denissen and Saguy, 2014; Willis, 2009). Importantly, harassment and microaggressions towards LGBT workers also serves a disciplining function; termed ‘gender policing’, abuse and aggression can be used to regulate binary gender norms (including heterosexuality), ‘establishing the boundaries of “normal” gender performance’ (Payne and Smith, 2016: 129). In the workplace, Willis (2009: 636) conceptualized this abuse as ‘reinforc[ing] the normalcy and “taken-for-grantedness” of heterosexuality in work cultures’. Despite this research, these findings have yet to be extended to include abuse and aggression from customers, instead focusing on relationships with co-workers and managers.

## Methods

Data for this study were collected as part of a community-engaged mixed methods study on the work, health and community experiences of LGBT workers in Windsor and Sudbury, Ontario (Mills et al., 2020). Windsor and Sudbury are two mid-sized Canadian cities—with populations of 329,144 and 164,689, respectively (Statistics Canada, 2017a, 2017b)—that are characterized by their industrial histories. Windsor, which is in the province’s south, has a history of automotive manufacturing that is continued today in the Chrysler and Ford plants that remain in operation. Sudbury, situated in the province’s northeast, is similar; while its economy was historically dominated by mining, the industry has slowly receded since the 1970s as work was made more precarious (King, 2017). Importantly, work in low-waged services has gained ascendency in both regions (Statistics Canada, 2017a, 2017b), which is particularly important to explore given that most research about LGBT workers has focused on white-collar workers in larger cities.

The study began with a community e-survey, administered between 6 July and 2 December 2018, that garnered 673 responses. Customer abuse and aggression emerged as a widespread issue facing LGBT workers: half of the 204 workers in the low-wage service sector reported experiencing abuse and/or aggression from customers, and workers who were transgender and/or racialized as people of colour reported customer abuse and aggression at significantly higher rates than those who were cisgender and/or racialized as white (Mills et al., 2020). As a result, the authors sought to explore customer abuse and aggression in the qualitative phase of the study. Participants were recruited using the e-survey itself, announcements and fliers at Pride festivities, social media and snowball sampling until a purposive sample was obtained that included representation across different gender identities, sexual orientations, racialized identities, ages and industries to capture the range of experiences included in the regions’ LGBT communities.

Of a total of 50 participants, 30 had recent and relevant experience working in the low-wage service sector. Of this group, 15 were transgender, six were Indigenous and two were racialized as people of colour, and participants ranged in age from 19 to 68. Face-to-face interviews were completed in Windsor and Sudbury from June to October 2019 and ranged from 60 to 120 minutes in length. Interviews asked about gender identity, sexual orientation and past and present work experiences, as well as views about union, employer and community supports. A semi-structured format allowed for in-depth discussions of experiences of abuse and aggression at work, including from customers. These interviews were then coded for experiences of customer abuse and aggression and analysed both inductively and deductively using NVivo 12, drawing on preestablished codes based on theory and early survey results while also allowing new themes to emerge during the coding process. Examples of customer abuse and aggression included a variety of material and symbolic manifestations of mistreatment, including microaggressions (or subtle forms of differential treatment and respect), discrimination, harassment and assault.

Interviews were conducted by four interviewers who shared experience with participants to different degrees. Two of the interviewers identified as lesbian and gay and one interviewer identified as transgender. Two of the interviewers had grown up in cities similar to those being studied and two interviewers were young people with recent experience working in low-wage services. Each interviewer was therefore able to move between insider and outsider status with each interviewee to varying degrees, sharing positionalities when possible to build participants’ trust (Dwyer and Buckle, 2009). Trust was also built through a community-engaged research process; researchers worked closely with community organizations, trade unions and worker centres and formed community advisory committees in both cities to advise on the interview protocol and recruit participants. Interviewers also spent time in the regions prior to interviews—meeting with community partners and attending Pride events—and drew on these shared experiences to build rapport. That said, the partial outsider status of the interviewers—for example, living in large urban centres and having higher incomes and educational attainment—may have affected participants’ openness, and consequently it is possible that the results underestimate experiences of customer abuse and aggression.

## Customer control and the gender and sexual expression of workers

Results build on previous studies showing how abuse and aggression can be used to discipline workers’ expressions of gender and sexuality. A small handful of studies have shown how abuse and aggression from employers and co-workers can help to enforce gendered and racial hierarchies critical to the labour process (Mezzadri, 2016; Salzinger, 2001). Salzinger (2001: 67) showed how sexual harassment and the conflation of ‘worker efficiency and desirability’ acted as a form of ‘sexualized surveillance’ of women workers to ensure that they met management’s expectations of femininity in an export-processing factory in Mexico. Mezzadri (2016) included sexual harassment as one of the ways that gendered commodification and exploitation were intertwined in the control of women garment workers in India by rendering them ‘disposable’. These examples, however, typically focus on the control of women’s bodies in workplaces clearly understood to be feminized. Building on scholarship about LGBT employment, this study demonstrates how understandings of cisnormativity and heteronormativity structure the labour process in an array of workplaces, including those that are not explicitly feminized.

Three types of narratives, together, demonstrate how customer abuse and aggression policed the gender and sexual expression of LGBT service workers. In some workplaces, managers communicated their desires for workers to express specific gender and sexual expression, setting the stage for customers to react aggressively when workers did not conform. Though in these cases customer abuse and aggression can be understood as a reaction to their unmet expectations, in practice it served as a mechanism of worker control that reinforced management prerogatives by amplifying pressure for workers to conform. Second, experiences of customer abuse and aggression often led to subjective forms of worker control as several workers described how past experiences of customer abuse and fears of future abuse and aggression compelled them to self-police their gender and sexual presentations by concealing non-conforming identities. For these workers, customer abuse and aggression had a more direct effect on their gender and sexual expression, contributing to the reproduction of cisgendered heterosexual norms believed to be desirable to customers. Last, the link between profitability, customer desires and gender and sexual expression was most clearly demonstrated in several cases where customers’ use of customer violence and aggression to control workers were tacitly supported by management non-interference. In these instances, management supported customers’ efforts to control workers—and even shifted their own expectations of workers’ gender expression—placing customer desires of normative gender and sexuality above LGBT workers’ safety.

Experiences of customer abuse and aggression were common among participants in the sample. Out of the 30 participants, 24 shared that they had experienced some form of abuse and/or aggression from customers and 14 shared experiences of microaggressions, seven shared experiences of verbal harassment, six shared experiences of discrimination, four shared experiences of sexual harassment, three shared experiences of sexual assault, and one shared an instance of physical assault. Data from the 10 participants who did not detail experiences of customer abuse and/or aggression also informed this study, with nearly all describing efforts to conceal their identities at work that may have protected against customer abuse and aggression.

### Reinforcing management’s control of worker appearance

Discrimination against workers on the basis of gender expression, identity and sexual orientation is unlawful in many jurisdictions, including Ontario. Since employers cannot legally discriminate or support harassment on these bases, more subtle mechanisms of control are required to maintain workplace norms. Many interview participants discussed pressures from management and customers to change their aesthetics and gender performance to ‘fit in’ at work, demonstrating how cisnormativity and heteronormativity were a valued component of the service interaction. LGBT workers explained how their employers conveyed the need to perform heterosexuality and cisgender normativity subtly through uniform requirements and tacit sanctions. Bailey^3^—a non-binary person—described: ‘I had to change my hair. I had to hide my tattoos. I had to dress in feminine clothes essentially.’ Uniform requirements were similarly restrictive for Florence, a pansexual woman. While the ways she presented as queer were officially condoned by management, she nonetheless experienced ‘harsher’ treatment based on this presentation: ‘Like they liked you less if you did [present differently] . . . you weren’t seen as the same level of professionalism as the other people if you were different like that’. As Filby (1992: 30) argues, management’s regulation of heterosexuality—and, by extension, normative gender performance—is rooted in a desire to mobilize sexuality ‘as a resource for commercial purposes’ and therefore enhance profitability.

Customers also participated in the policing of gender and sexual norms at work, and customer abuse and aggression experienced by LGBT workers often worked in consort with management to uphold normative gender and sexual expression. In cases where workers did not—or could not—change their presentation or performance, customers exerted pressures to conform through abuse and aggression. This had particularly adverse effects for transgender workers. At Marcus’s workplace, aesthetic requirements were strict and followed a binary gendered dress code. Women were expected to wear a specific number of makeup products on their face, and management was resistant when Marcus—a transgender man—asked to switch to the men’s dress code. Transgender workers who did not pass challenged this binary. When Marcus revealed his gender identity to a customer, she became hostile and no longer wanted his service:

She threw a fit [. . .] she just didn’t want me touching her anymore. She had been totally fine with the [job] before. She loved how it was going or whatever. And then yeah she did not want me touching her. She said, ‘This is why people like you should not be allowed to . . .’, like you should be locked up essentially.

While the customer’s abuse and aggression was structured by the workplace’s aesthetic requirements that reinforced normative expectations of gender and sexuality and eroded the separation between worker and product, such that ‘consumers may perceive the worker as part of the service’, it also communicated the customer’s desire to be served by cisgender employees (Ryan-Flood, 2004: 11). This desire for workers to present as cisgender was also communicated by a manager who then retreated when Marcus cited the employee handbook:

One time after I’d come out, I did come into work without makeup on [. . .] I wasn’t wearing mascara, but I was wearing other things. Mascara was something that made me feel pretty dysphoric [. . .] as soon as I had like long eyelashes I was like ‘that’s too feminine, I look like a chick’, [. . .] but a manager who doesn’t work there anymore said like, ‘You’re supposed to be wearing mascara’. And I said, ‘It says in the handbook that male employees don’t have to’, and he said, ‘Well, we’ll have to like double check on that’.

Insofar as the desires of the customer to be served by a cisgender employee were shared by management, Marcus’s experience can be read both as an example of customer abuse bolstering management’s attempts to control worker aesthetics and as the reaction to a gendered labour process, a rejection of a product that did not meet the customer’s gendered expectations set by the organization itself. While expressing his identity as a transman was not outside of formal workplace policies, it clearly violated the informal norms and expectations of the workplace for workers to present as cisgender and gender normative. In this setting, customers’ abuse and aggression worked with dress codes and other workplace norms to define and control how workers expressed their gender.

Florence, a pansexual woman, recounted how customers often communicated this form of ‘rejection’ non-verbally. After describing a series of instances where she was misgendered due to her non-normative, queer gender presentation, she explained how customers often ignored her and sought service from others:

I don’t know if it’s because I was annoying or if it’s because of how I presented myself, but they would definitely try to ignore me the best that they could [. . .] Yeah, or like scoff a little bit.

These small acts carried symbolic weight that affected Florence’s comfort with customers and in the workplace. The act of ‘ignoring’ can communicate to LGBT workers that they are unwelcome, adding to a sense of exclusion that already pervades heteronormative environments (Willis, 2009). Research has also found higher rates of hostility against non-conforming women, suggesting that individuals who downplay femininity are at a greater risk for mistreatment (Gordon and Meyer, 2007).

These examples demonstrate how workers faced abuse and aggression from employers and customers when they did not conform to heterosexual and cisgender norms. Participants were not only expected to adjust their dress to conform to gender norms; other aspects of their presentation and performance, including body modifications and mannerisms, were also the object of control. Similar to Warhurst and Nickson’s (2009) findings, workers’ sexuality and appearance were mobilized by management as a means of attracting customers and were realized through workers’ aesthetic labour. Importantly, sexuality norms are premised on heterosexuality, and ‘constitute part of the “ideal/model” employee’ in service workplaces (Ryan-Flood, 2004: 11). While incidents of customers’ abuse and aggression were influenced by the gendered labour process of the workplace, they were also attempts by the customer to exercise aesthetic control over workers that ultimately helped to reinforce these gendered norms.

### Concealment and customer abuse and aggression

Customer abuse and aggression was also intimately connected to participants’ decisions to disclose or conceal their sexual and/or gender identities at work, becoming a form of normative control by encouraging workers to conform to the gender and sexuality ideals of their workplaces to avoid conflict and fit in. For many, concealment was intimately connected to past experiences and fears of customer abuse and aggression, demonstrating how customer abuse and aggression can lead to self-policing. John, a gay man, described this when discussing his gender performance with customers: ‘I try to reel in my mannerisms a little bit when I’m at work just for the sake of avoiding conflict, but when we’re having fun sometimes it comes out’. As a non-binary person working in fast food, Parker felt like they were ‘playing a part’ at work—something they also described as ‘exhausting’. When asked whether they would find being out at work liberating, fear of abuse outweighed this goal: ‘I’d be too scared because I feel like certain people would follow me into the parking lot’. Caitlin, a bisexual woman who was concerned about customers because she had recently cut her hair short, connected pressures to conceal to her remuneration: ‘I’m worrying I’m not going to get the job, that I’d get less tips and that people would be able to clock me as being queer [. . .] they can’t really tell what’s going on because I still dress very feminine’.

For participants who did or could not conceal their identities, some instances of customer abuse and aggression clearly targeted their sexual orientation or gender identity, exemplifying the ‘conflict’ that John desired to avoid. Prior to his transition, Kyle experienced sexual assault based on his sexual orientation—which he, at the time, identified as lesbian—from customers who thought they could ‘change’ him:

I think, also, some of the guys were even more harassing because I identified as preferring women, because they thought they could change me. Yeah, I think, at least personally, I think they were a little more insistent because of it. And there was no help from management or anyone [. . .] So, like I have had my ass grabbed and told ‘I can turn you back onto [men]’.

In response to this abuse, Kyle tried to conceal his gender identity and sexuality by ‘hyper-feminizing’ his gender performance, wearing heels, makeup and corsets to fit the expectations of the establishment he worked at. This process of self-policing—spurred by customer abuse and fears thereof—encouraged Kyle to reproduce gender and sexuality norms by changing his presentation, something he described as ‘exhausting’. After transitioning, Kyle could no longer conceal his trans identity and experienced abuse: ‘I’ve been called “tranny trash” now twice’.

Customer abuse and aggression was often misogynistic and denigrated non-cis and heterosexual identities, reinscribing the gender hierarchy and dominance of heterosexuality in the workplace and signalling a desire to be served by heterosexual women (Giuffre et al., 2008; Willis, 2009). By troubling the dominance of heterosexual men, the identities of sexual and gender minorities became an arena of contestation between workers and customers, one that customers sought to discipline and control through abuse and aggression. This control was then internalized by workers, who engaged in self-policing by concealing their identities and changing their presentation to avoid future conflict with customers.

### Management, profit and customer sovereignty

Customer efforts to control the service interaction through customer abuse and aggression were viewed by managers through a lens of profitability. Efforts by customers to uphold gender and sexual norms were particularly effective when they were tacitly sanctioned by management allowing customers to control workers’ behaviour more directly. In several cases, management did not intervene to stop abuse or aggression, allying with customers against workers to maintain customer sovereignty—or the ‘illusion’ that the customer is king (Bishop and Hoel, 2008).

Decisions about whether to support workers and intervene in customer abuse and aggression were often based on the profitability of the interaction and were made at the expense of LGBT workers’ safety. For Marcus, a transgender man who worked in retail and described how he did not always pass with customers, this was particularly clear. Describing how management reacted to customers who were verbally and physically abusive, he said:

It really depends on how much money they’re spending, which is sad. If it’s like some person that they think is, you know, on drugs or just here to get samples or whatever, or they’ve never seen them before or if it’s like a teenage boy, they’ll just kick them out [. . .] if it’s somebody who, say, is a big name in the community or like they come in and they blow a thousand dollars every month . . . they’ll just tell me to go to the back and distract myself through other work.

Key here are the ways Marcus was directed to respond to profitable customers. Instead of confronting these customers when they were abusive towards LGBT workers—something which would dispel the notion of customer sovereignty—management would remove Marcus from the interaction and replace him with another worker who could fulfil customers’ desires for gender and sexual expression and complete the sale. This physical removal additionally curtailed Marcus’ capacity to resist the service interaction.

Kyle had a similar relationship with management. As a transgender man working in food service who experienced considerable discrimination from customers, his managers provided support in some instances and upheld the sovereignty of customers in others, protecting his safety only when doing so had a limited effect on business. When management overheard Kyle being verbally abused and repeatedly misgendered by an individual customer, their response was swift:

Yesterday I had a customer yelling at me because her husband made a mistake when ordering [. . .] [My manager said] ‘What are you fucking talking about? My employee is a he.’ [And then the customer said] ‘Tranny trash’. [And my manager said] ‘I’ll happily refund your ticket now’.

Alternatively, when intervening jeopardized the patronage of multiple customers, the manager sided with customers, going so far as to change workplace policy and accommodate explicit discrimination by forbidding him from using the men’s bathroom:

I’ve had people complain to my boss about me using the men’s bathroom to the point where I’m only allowed to use the official staff bathroom, because there’s been too many complaints.

In addition to exerting control over Kyle by preventing him from using the men’s bathroom—a form of discrimination—customer feedback through indirect abuse and aggression also resulted in management’s decision to prescribe cisnormativity in the workplace. Indeed, customers’ discriminatory complaints changed the ‘gender rules’ enforced by management, who accommodated customers’ desires for greater gender and sexual normativity to maintain profitability.

These dynamics were also implicated in participants’ experiences of sexual harassment. For John, a gay man who worked in entertainment, sexual harassment towards him and his colleagues was ‘extremely common’ and connected to his sexual orientation. He often had conflict with management over whether to involve police, since management felt it would hurt profits:

There is an issue with [sexual harassment] and it’s sometimes I have to get the police involved but the management try to tell you not to because you’re taking away from their [customer] base, so it’s a very difficult atmosphere to navigate basically if you identify as anything even remotely as a minority.

In these interactions, management decisions to uphold customer sovereignty instead of intervening helped to cement cisnormativity and heteronormativity in the workplace and strengthened customers’ ability to exert control over LGBT workers through customer abuse and aggression. Customer sovereignty and normative gender and sexuality are both intimately connected to profit, too, as the former preserves the ‘enchanting’ elements of consumption while shepherding the customer through a rationalized labour process (Korczynski and Ott, 2004), and the latter draws upon sexualized aesthetics to attract customers (Warhurst and Nickson, 2009). When management refrained from intervention or shifted the parameters of what was acceptable to maximize profit, abuse and aggression that targeted and controlled LGBT workers in the workplace was thus tacitly sanctioned, upholding gender hierarchies and the devaluation of non-heterosexual identities in service workplaces.

## Conclusion and discussion

LGBT workers’ experiences of customer abuse and aggression rendered visible the cis and heteronormativity of the labour process in the low-wage service sector while demonstrating the influence of customers over this process. These findings contribute to previous research on customer abuse and aggression in two ways. First, LGBT workers’ experiences of customer abuse and aggression inform understandings about how gender/sexual expression operates alongside gender and race in service labour processes. Second, results support the argument that customer abuse and aggression is not simply a reaction to the service labour process, but also a mechanism of control over workers and the service interaction as a whole.

Previous research has shown how service workplaces often demand performances of heterosexuality and femininity that increase worker vulnerability to customer abuse and aggression (Folgerø and Fjeldstad, 1995; Forseth, 2005; Warhurst and Nickson, 2009). In this study, workers were pressured by customers and managers to conform not only to femininity and heterosexuality, but also to cisnormativity—a phenomenon that has adverse consequences for many LGBT workers, particularly those who identify as transgender. While some workers concealed their identity or altered their gender and sexual expression to avoid conflict with customers and management, others were unable to do so. Transgender and LGB workers were particularly vulnerable to abuse and aggression both because of their membership in a denigrated group, and because they challenged the gender and sexual norms desired by customers (Korczynski and Evans, 2013; Warhurst and Nickson, 2009).

These results extend research about ‘gender policing’ to the realm of customer service, which scholars have already shown to be imbued with gendered cultural expectations (Ryan-Flood, 2004; Warhurst and Nickson, 2009). Research about women in male-dominated trades found that workers—particularly women—experienced abuse when they were unable to meet the culturally embedded gendered expectations of male co-workers (Denissen and Saguy, 2014; Paap, 2006). Indeed, denigrating those who do not display required masculinities allows men to position themselves as more masculine and hence as more highly skilled, and encourages the adoption of behaviours and presentations that align with the gender and sexual expressions and behaviours rendered ‘acceptable’ in a given context (Payne and Smith, 2016; Willis, 2009). In this study, LGBT workers engaged in low-wage service work experienced gender policing even in workplaces that were not explicitly gendered, underscoring the extent to which cisnormativity is embedded in the service labour process as a desired aesthetic from customers and management.

This article also extends previous research about contested power and control in the service triad by showing how customers and managers often have a shared interest in constraining workers’ gender and sexual expression. Customer abuse and aggression worked in support of management’s efforts to constrain workers’ gender and sexual expression and also independently. Customer abuse and aggression variably served to: reinforce management’s desires to maintain cisnormative and heterosexual gendered aesthetics; directly influence worker behaviour by causing them to conceal their identities; and help set gender norms in the workplace by influencing management decisions about what customer and worker behaviour was acceptable. While managers occasionally contested customers’ behaviour and supported LGBT workers against customers, responses were dictated by the profit imperative, and abuse and aggression was often tacitly supported. This foregrounds the profitability of both customer sovereignty and cisnormative gender expression in the service sector. Indeed, customer efforts to control the gender and sexual norms of the service experience were more successful when their interests complemented those of management. When managers upheld ideas of customer sovereignty in the face of customer abuse and aggression, the profitability of normative gender and sexuality was put above the safety of LGBT workers.

In the context of multiple pressures to present normative gender and sexual identities, being ‘out’ about transgender and non-heterosexual identities can be understood as an individualized form of resistance. Though being ‘out’ at work might render workers more vulnerable to abuse and aggression, it also acts against the gender and sexual normativity of the workplace. More research is warranted to understand if LGBT workers find collective ways of resisting customer abuse and aggression, such as forming communities of coping (Korczynski, 2003).

## Notes

1. In this article, the term ‘LGBT’—the acronym for lesbian, gay, bisexual and transgender—is used to include all sexual and gender minorities, including those who identify as Two-Spirit, asexual and queer.

2. In 2019, 79% of Canada’s employment was in the service sector, with 21.3% in the low-wage sectors of accommodation and food services and wholesale and retail trade. Goods producing sectors comprised 20.8% of employment (Statistics Canada, 2019).

3. To protect participants’ identities, all participants were given pseudonyms.
